# Systolic MOLLI T1 mapping with heart-rate-dependent pulse sequence sampling scheme is feasible in patients with atrial fibrillation

**DOI:** 10.1186/s12968-016-0232-7

**Published:** 2016-03-15

**Authors:** Lei Zhao, Songnan Li, Xiaohai Ma, Andreas Greiser, Tianjing Zhang, Jing An, Rong Bai, Jianzeng Dong, Zhanming Fan

**Affiliations:** Department of Radiology, Beijing Anzhen Hospital, Capital Medical University, 100029 Beijing, China; Department of Cardiology, Beijing Anzhen Hospital, Capital Medical University, Beijing, China; Siemens Healthcare, Erlangen, Germany; MR Collaborations NE Asia, Siemens Healthcare, Beijing, China

**Keywords:** T1 mapping, Modified Look-Locker inversion recovery, Extracellular volume fraction, Atrial fibrillation, Cardiovascular magnetic resonance

## Abstract

**Background:**

T1 mapping enables assessment of myocardial characteristics. As the most common type of arrhythmia, atrial fibrillation (AF) is often accompanied by a variety of cardiac pathologies, whereby the irregular and usually rapid ventricle rate of AF may cause inaccurate T1 estimation due to mis-triggering and inadequate magnetization recovery. We hypothesized that systolic T1 mapping with a heart-rate-dependent (HRD) pulse sequence scheme may overcome this issue.

**Methods:**

30 patients with AF and 13 healthy volunteers were enrolled and underwent cardiovascular magnetic resonance (CMR) at 3 T. CMR was repeated for 3 patients after electric cardioversion and for 2 volunteers after lowering heart rate (HR). A Modified Look-Locker Inversion Recovery (MOLLI) sequence was acquired before and 15 min after administration of 0.1 mmol/kg gadopentetate dimeglumine. For AF patients, both the fixed 5(3)3/4(1)3(1)2 and the HRD sampling scheme were performed at diastole and systole, respectively. The HRD pulse sequence sampling scheme was 5(n)3/4(n)3(n)2, where n was determined by the heart rate to ensure adequate magnetization recovery. Image quality of T1 maps was assessed. T1 times were measured in myocardium and blood. Extracellular volume fraction (ECV) was calculated.

**Results:**

In volunteers with repeated T1 mapping, the myocardial native T1 and ECV generated from the 1^st^ fixed sampling scheme were smaller than from the 1^st^ HRD and 2^nd^ fixed sampling scheme. In healthy volunteers, the overall native T1 times and ECV of the left ventricle (LV) in diastolic T1 maps were greater than in systolic T1 maps (*P* < 0.01, *P* < 0.05). In the 3 AF patients that had received electrical cardioversion therapy, the myocardial native T1 times and ECV generated from the fixed sampling scheme were smaller than in the 1^st^ and 2^nd^ HRD sampling scheme (all *P* < 0.05). In patients with AF (HR: 88 ± 20 bpm, HR fluctuation: 12 ± 9 bpm), more T1 maps with artifact were found in diastole than in systole (*P* < 0.01). The overall native T1 times and ECV of the left ventricle (LV) in diastolic T1 maps were greater than systolic T1 maps, either with fixed or HRD sampling scheme (all *P* < 0.05).

**Conclusion:**

Systolic MOLLI T1 mapping with heart-rate-dependent pulse sequence scheme can improve image quality and avoid T1 underestimation. It is feasible and with further validation may extend clinical applicability of T1 mapping to patients with atrial fibrillation.

## Background

Atrial fibrillation (AF) is the most common clinical cardiac arrhythmia. The mechanisms of AF are complex and associated with structural and electrical remodeling in the atrial and ventricular myocardium [1]. Development of atrial fibrosis is the hallmark of structural remodeling in AF and considered the substrate for AF. In contrast, data on ventricular fibrosis of patients with AF are limited, although ventricular fibrosis has a detrimental effect on both systolic and diastolic function in patients with AF [2, 3]. Atrial and ventricular fibrosis in AF is likely to share many common mechanisms, including excessive accumulation of collagen either in regions of myocyte loss or diffusely in areas of extracellular matrix in the interstitium [4]. T1 mapping and derived extracellular volume fraction (ECV) enable quantitative assessment of myocardial characteristics, such as edema, focal and diffuse fibrosis [5–7]. T1 mapping and ECV may provide useful information for patients with AF. However, the irregular and usually rapid ventricular rate hindered the application of T1 mapping in patients with AF. Although different T1 mapping methods such as saturation-pulse-prepared heart-rate-independent inversion recovery T1 mapping have potential advantages in arrhythmia [8], the Modified Look-Locker Inversion Recovery (MOLLI) sequence is still widely used due to its higher precision and better reproducibility [9, 10]. The heart rate sensitivity of MOLLI has been significantly reduced by modification of sampling schemes (5(3)3 and 4(1)3(1)2 for pre-contrast and post-contrast T1 mapping, respectively) to the point where it is of much less concern [11]. The largest factor that affects the MOLLI heart rate sensitivity was the time between the inversions. It can be mitigated by increasing the time between inversions [12]. But the exact method how to increase the time between inversions is not well established yet. In addition, at higher heart rate, the time window with minimal motion in diastole shortens more than in systole. In patients with AF, although the R-R interval varies in each cardiac cycle, the variation in systole is smaller than in diastole [11]. It is potentially practicable to acquire T1 mapping in systole in patients with AF.

Therefore, we hypothesize that systolic T1 mapping with heart-rate-dependent (HRD) pulse sequence sampling scheme may be feasible in patients with AF. We compared the differences of T1 times and ECV acquired in systolic and diastolic phase with fixed/HRD pulse sequence sampling scheme, and assessed the accuracy of systolic T1 mapping with HRD pulse sequence sampling scheme in patients with AF who underwent cardiovascular magnetic resonance (CMR) twice, before and after electric cardioversion.

## Methods

The study was approved by Beijing Anzhen Hospital ethics committee, and written informed consent was obtained from all participants. A detailed explanation concerning contrast agent was given to each participant.

### Study population

Thirteen healthy volunteers and thirty patients with “persistent” AF without contraindication to CMR were recruited. None of the healthy volunteers was referred as patient for clinical CMR which then turned out to be normal, all of them had no evidence or risk factors of cardiovascular disease. The AF patients were defined as “persistent”, whose duration of continuous AF episodes lasted > 7 days or required electrical cardioversion. Exclusion criteria were presence of a permanent pacemaker, severe claustrophobia, and severe impairment of renal function (glomerular filtration rate < 30 mL/min/1.73 m^2^).

### HRD pulse sequence sampling scheme determination

In the MOLLI sequence with fixed sampling scheme, such as 5(3)3 or 4(1)3(1), the inversion time between data acquisition is fixed as 3 heartbeats or 1 heartbeat as shown in the parentheses. At higher heart rate, each single heartbeat duration is shorter than at slow heart rate. Under such circumstances, the shorter inversion time will lead to inadequate magnetization recovery before acquiring the next data inversion episode, which results in underestimation of T1 times. To ensure the full recovery of longitudinal magnetization before the following inversion pulse is applied, the following pause in units of heartbeats needs to be increased along with the increase of the heart rate. Theoretically, after a duration of 4 times of T1 time (the maximal T1 time expected for the tissue of interest is approximately 2000 ms), the magnetization of tissue is almost back to equilibrium. For the 5(3)3 scheme, at heart rate of 60 beats per minute (bpm, RR interval = 1000 ms), it takes (5 + 3) × 1000 = 8000 ms (which equals to 4 times of tissue of interest’s T1 time) until the second inversion is played out. At higher heart rate, using the above scheme may lead to inadequate inversion recovery. It would make sense to set up different sampling schemes that adapt to the increased heart rate.

**Fig. 1:**
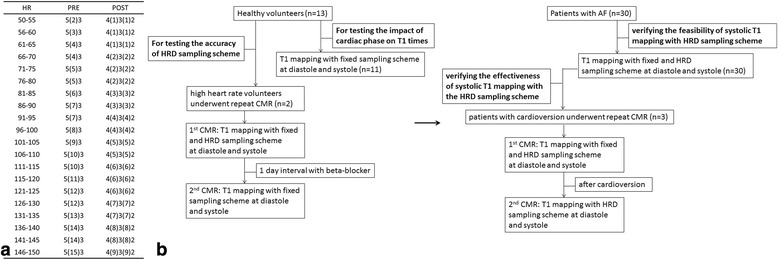
Flow chart and heart-rate-dependent sampling scheme. **a**, the heart-rate-dependent sampling schemes for heart rate ranges with bpm increment of 5 from 50 to 150 bpm; **b**, flow chart of study, first performed in healthy volunteers (left), then in patients with AF (right). HR = heart rate; HRD = heart rate dependent; CMR = cardiovascular magnetic resonance

For the consideration of ensuring complete magnetization recovery at higher heart rates, the sampling schemes 5(3)3 and 4(1)3(1)2 were adopted to modify the recovery to be determined by heart rate. Based on the principle that 5(3)3 scheme is optimized for a heart rate of 60 bpm (1000 ms), for the pre-contrast T1 mapping, the heartbeat number of the second T1 inversion recovery is calculated as: n = (5+3)/(heart rate in ms) × 1000-5; for the post-contrast T1 mapping, the heartbeat number of the inversion is calculated as: n = (4+1)/(heart rate in ms) × 1000-4. We set up sampling schemes for various heart rate ranges with bpm increment of 5 from 50 to 150 bpm (Fig. 1a).

### Study procedure

For the purpose of testing the feasibility and accuracy of T1 mapping with the HRD pulse sequence sampling scheme, two healthy volunteers in normal sinus rhythm with relatively high heart rate (HR, volunteer 1: 67–69 bpm, volunteer 2: 86–89 bpm) during first scan were asked to undergo two CMRs before and after taking beta-blockers. They underwent T1 mapping with fixed and HRD sampling scheme at first scan, and at the next day, after taking beta-blocker, they underwent the second T1 mapping scan with fixed sampling scheme (HR during scan: 57–59 bpm and 58–60 bpm, respectively). The left-ventricle myocardial per-segment native T1 time and ECV generated from fixed sampling scheme at first scan, HRD sampling scheme at first scan, and fixed sampling scheme at second scan were compared.

For observing the impact of different cardiac phases (systole and diastole) on T1 times, 11 healthy volunteers in normal sinus rhythm (HR: 60 ± 5 bpm, HR fluctuation: 3 ± 3 bpm) underwent T1 mapping with fixed sampling scheme (5(3)3/4(1)3(1)2) at diastole and systole. The number of T1 maps and myocardial segments with artifact generated from different cardiac phases were recorded and compared; the T1 time and ECV generated from different cardiac phases were compared.

For verifying the feasibility of systolic T1 mapping with HRD sampling scheme, patients with AF (HR: 88 ± 20 bpm, HR fluctuation: 12 ± 9 bpm) underwent T1 mapping with fixed and HRD sampling scheme at diastole and systole. The number of T1 maps and myocardial segments with artifact generated from different sampling scheme and cardiac phases combination were recorded and compared; the T1 time and ECV generated from different sampling scheme and cardiac phases combination were compared.

For the purpose of verifying the accuracy and effectiveness of systolic T1 mapping with the HRD pulse sequence sampling scheme, three patients (with AF) who received electric cardioversion, at the next day of cardioversion underwent the second T1 mapping with HRD sampling scheme in sinus rhythm, at diastole and systole. The left-ventricle myocardial per-segment native T1 time and ECV generated from different sampling schemes and cardiac phases combination at first scan, and HRD sampling scheme with different cardiac phases at second scan were compared. The flow chart of study is showed in Fig. 1b.

### CMR acquisition

All CMR was performed using a 3 T MR system (MAGNETOM Verio, Siemens Healthcare, Erlangen, Germany) with a 32-channel cardiac coil. Subject-specific, volume-selective first- and second-order B0-shimming based on field maps derived from double-gradient-echo acquisitions was performed to improve static field uniformity.

T1 mapping sequence was obtained using a Siemens prototype (#448B, system software version *syngo* MR B17A). Data were acquired in basal, mid-ventricular, and apical short-axis planes at diastole and systole before and 15 min after administration of 0.1 mmol/kg gadopentetate dimeglumine (Magnevist, Bayer Healthcare). The diastolic phase was determined by the system with captured trigger delay (TD) according to the ECG gating, the systolic phase was set to TD of 0 ms (the acquisition started after 120 ms due to the TI start time) [13]. For the HRD sampling scheme, the number of inversions was determined by referencing the highest heart rate before the actual scan. Imaging parameters were: TR = 2.6-2.7 ms, TE = 1.0-1.1 ms, FA = 35°, FOV = (270 × 360) mm^2^, matrix = 256 for heart rate < 90 bpm, 192 for heart rate > 90 bpm, slice thickness = 6 mm, BW= 1045-1028Hz/px, GRAPPA acceleration factor 2, linear phase-encoding ordering, minimum TI of 120 ms. The quality control was performed during scanning by reviewing the “goodness of fit” map and source images to allow an immediate repetition of suboptimal measurements to minimize the respiratory motion and off-resonance effects. The blood sample was taken just before CMR to measure hematocrit for ECV calculation.

For the purpose of clinical diagnosis, all patients also received CMR protocols including cine and late gadolinium enhancement (LGE) imaging as described elsewhere [14].

### Image analysis

All image datasets were transferred to a workstation (Viewing and Argus, Siemens Healthcare, Erlangen, Germany) for offline analysis. The T1 maps and source images were assessed, myocardial segments with artifact were excluded for further analysis. Two experienced readers assessed artifacts in consensus. The heart rate of each cardiac cycle during scanning of every image was recorded, the variability in heart rate was calculated as the standard deviation from the mean heart rate.

The left-ventricular (LV) myocardium was delineated by manually contouring the endo-cardial and epi-cardial borders. Care was taken to avoid contamination of signal from blood or epi-cardial fat. T1 time of the blood was measured by manually drawing a region of interest in the LV cavity of T1* map (T1 map based on fitting without Look-Locker correction) taking care to avoid the papillary muscles. When artifact existed in certain segment in image originated from any single diastole/systole and fixed/HRD sampling scheme combination, the patient’s same segment generated from T1 maps with other different cardiac phase and sampling scheme combinations were excluded for per-segment pairwise comparison. But all of the assessable segments generated from any single diastole/systole and fixed/HRD sampling scheme combination were included in calculating the respective average overall LV myocardial T1 times. The overall LV myocardial native T1 time was mean of T1 times of basal, mid-ventricular and apical levels. For the participants with repeated CMRs, all of the pre- and post-contrast per-segment (according to American Heart Association myocardial 17 segments classification, apical segment was excluded in this analysis) myocardial T1 times were drawn in T1 maps generated from different cardiac phases and sampling scheme combinations. ECV was calculated from T1 maps acquired pre- and post-contrast calibrated by blood hematocrit [15]. The ECV was calculated as:

$$ ECV=\left(1- hematocrit\right)\left(\frac{1}{T1myo\kern.3em  post}-\frac{1}{T1myo\kern.3em pre}\right)/\left(\frac{1}{T1 blood\kern.3em  post}-\frac{1}{T1 blood\kern.3em pre}\right) $$

### Statistical analysis

Data are presented as mean and standard deviation. Differences between means were tested by the paired *t*-test and one-way analysis of variance as appropriate. The assessable images and segments were compared between systole and diastole using *χ*^2^ test. The overall LV myocardial native T1 times and ECV generated from different cardiac phases and sampling scheme combinations were compared in all participants. The per-segment myocardial native T1 times and ECV generated from different cardiac phases and sampling scheme combinations were compared in participants with repeated CMRs. The statistical significance was defined as *p* < 0.05. Statistical analysis was performed using SPSS software (SPSS Inc., Chicago, IL, USA, version 17.0).

## Results

The demographic data, heart rate and heart rate fluctuation during scan, LV functional indices of healthy volunteers and patients with AF are listed in Table 1.

**Table 1:** Demographic data and left-ventricle functional indices of healthy volunteers and patients with atrial fibrillation

	Healthy volunteers (*n* = 13)	Patients with AF (*n* = 30)
Gender, female	3 (23.08 %)	10 (33.33 %)
Age (years)	53 ± 18	54 ± 13
Height (m)	1.70 ± 0.06	1.69 ± 0.09
Weight (kg)	76.35 ± 14.97	76.20 ± 15.21
BMI (kg/m^2^)	26.17 ± 3.87	26.46 ± 3.94
Average HR (bpm)	62 ± 9	88 ± 20
HR fluctuation (bpm)	3 ± 3	12 ± 9
Left atrium volume (cm^3^)	139.03 ± 45.05	158.42 ± 50.55
EF (%)	63.17 ± 7.00	49.19 ± 11.83
EDV (ml)	98.17 ± 19.96	87.81 ± 26.85
ESV (ml)	36.46 ± 10.96	44.94 ± 19.51
SV (ml)	61.71 ± 13.13	42.89 ± 16.61
MM (g)	86.12 ± 24.59	85.94 ± 25.01
CO (l/min)	3.97 ± 1.17	3.58 ± 1.10
LGE positive subject	0	10

Data were presented in mean ± standard deviation. *AF* atrial fibrillation, *BMI* body mass index, *HR* heart rate, *EF* ejection fraction, *EDV* end-diastolic volume, *ESV* end-systolic volume, *SV* stroke volume, *MM* left-ventricle myocardial mass, *CO* cardiac output, *LGE* late gadolinium enhancement

### Comparison between HRD and fixed sampling scheme in normal sinus rhythm with high heart rate

For the two healthy volunteers that underwent two CMRs, volunteer 1 presented HR of 67–69 bpm during the first scan, the HRD sampling scheme was 5(4)3/4(2)3(2)2, volunteer 2 presented a HR of 86–89 bpm during the first scan with HRD sampling scheme of 5(7)3/4(3)3(3)2. In volunteer 1, there were no significant differences of per-segment myocardial native T1 times and ECV among 1^st^ 5(3)3/4(1)3(1)2, 1^st^ 5(4)3/4(2)3(2)2 and 2^nd^ 5(3)3/4(1)3(1)2 either in diastolic or systolic T1 maps (all *p* > 0.05). In volunteer 2, there were significant differences of per-segment myocardial native T1 times and ECV between 1^st^ 5(3)3/4(1)3(1)2 and 1^st^ 5(7)3/4(3)3(3)2, and between 1^st^ 5(3)3/4(1)3(1)2 and 2^nd^ 5(3)3/4(1)3(1)2 either in diastolic or systolic T1 mappings (all *p* < 0.05); but no significant differences of per-segment myocardial native T1 times and ECV between 1^st^ 5(7)3/4(3)3(3)2 and 2^nd^ 5(3)3/4(1)3(1)2 either in diastolic or systolic T1 mappings (all *p* > 0.05).

### Comparison between systole and diastole T1 mapping in healthy volunteers

In the 11 healthy volunteers, there were 66 myocardial native T1 maps and 66 myocardial post-contrast T1 maps (11 volunteers × 3 slices × 2 (1 of diastole and 1 of systole)). 6 segments in 2 native T1 maps and 7 segments in 3 post-contrast T1 maps were excluded due to the presence of artifacts. In the native T1 maps with artifacts, 4 segments of 1 T1 map were diastolic images; in the post-contrast T1 maps with artifacts, 5 segments of 2 T1 maps were diastolic images. There were no significant differences of assessable images between systole and diastole (*χ*^2^ = 0.204, *p* > 0.05).

The LV overall native T1 times, post-contrast T1 times and ECV of myocardium, pre-/post-contrast blood T1 times of diastolic and systolic T1 mapping were compared and are presented in Table 2. There were minor but statistically significant differences in myocardial and blood T1 times and ECV between diastolic and systolic T1 maps.

**Table 2:** Comparison of pre-/post-contrast myocardium and blood T1 times and ECV between diastolic and systolic T1 maps in healthy volunteers

	Diastole	Systole	*P* value
Overall LV myocardium native T1 times (ms)	1247.73 ± 31.86	1231.06 ± 31.45	<0.01
Overall LV myocardium post-contrast T1 times (ms)	544.94 ± 52.80	563.80 ± 56.89	<0.01
Overall LV pre-contrast blood T1 times (ms)	1832.76 ± 61.76	1784.49 ± 82.02	<0.05
Overall LV post-contrast blood T1 times (ms)	352.87 ± 49.15	365.53 ± 51.70	<0.01
Overall LV ECV (%)	25.11 ± 1.63	24.60 ± 1.58	=0.047

Data are presented in mean ± standard deviation. *LV* left ventricle, *ECV* extracellular volume

### Comparison among different HRD/fixed sampling schemes and systole/diastole combination T1 mapping in patients with AF

**Fig. 2:**
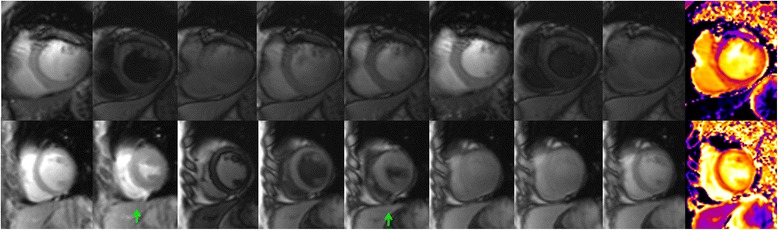
Artifacts of T1 mapping images. Upper line are the example images output by T1 mapping illustrate the off resonance related dark banding artifact, the line-like artifact at anterior segment are presented in every source image and color map; lower line are the example of mistrigger related motion artifact, all images except the 2^nd^ and 5^th^ image (green arrow) were acquired at diastolic phase, the inconsistent contour of the source images lead to the blur appearance of the final color map

**Fig. 3:**
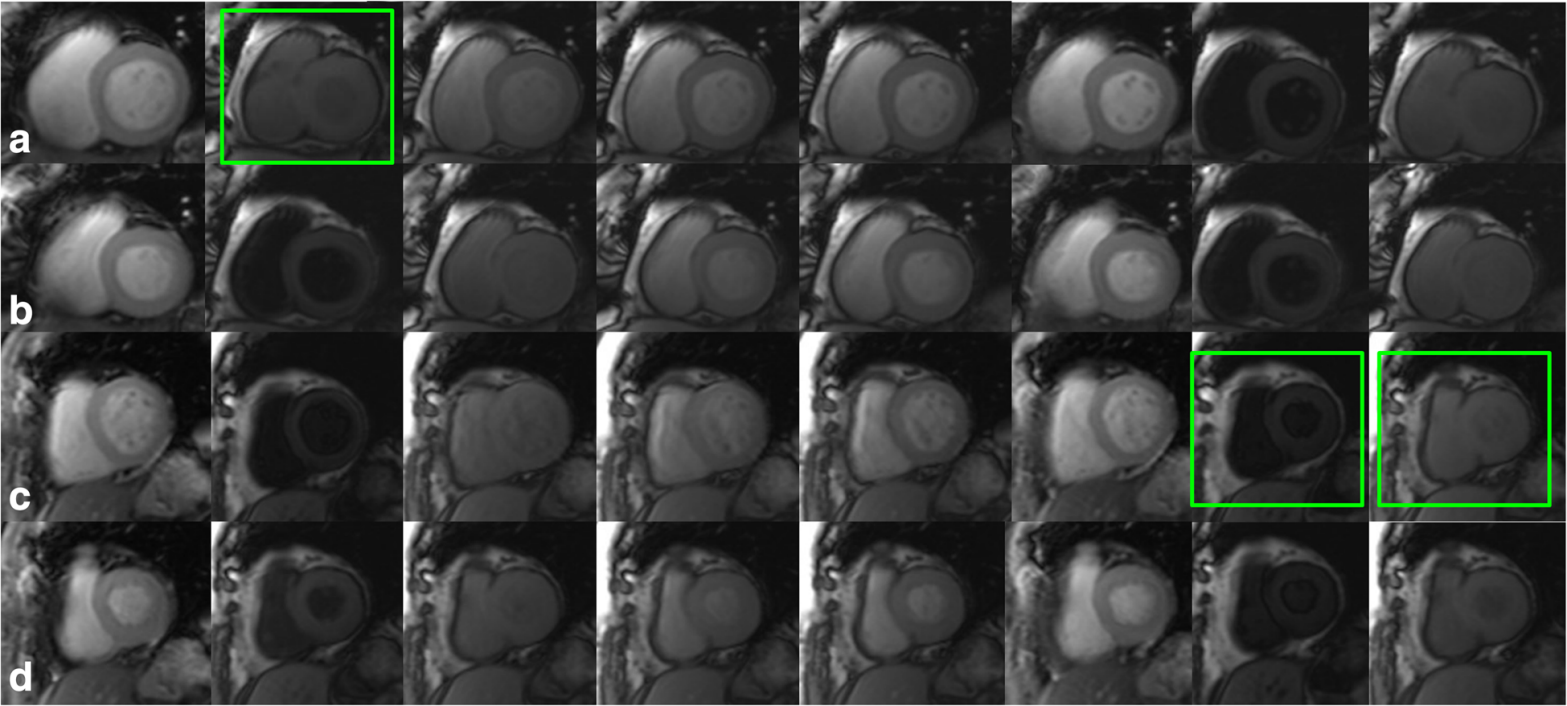
Comparison of diastolic and systolic T1 mapping images in patients with AF. Example images output by T1 mapping in patients with AF. **a**-**b**: a AF patient with heart rate ranged from 54 to 74 bpm during scan. **a** presented the motion corrected IR images acquired at system default diastolic trigger delay, the second image (as shown in the green box) was mistriggered at systolic phase; **b** presented the motion corrected IR images acquired at systolic phase, the images are in consistent contour. **c**-**d**: a AF patient with heart rate ranged from 72 to 112 bpm. **c** presented the motion corrected IR images acquired at system default diastolic phase, the 7^th^ and 8^th^ images (marked with green box) were mistriggered at systolic phase; **d** presented the motion corrected IR images acquired at systolic phase, no mistriggered images was found

**Fig. 4:**
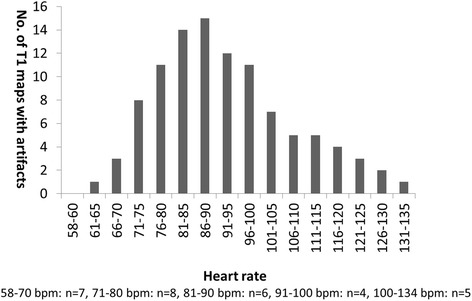
Numbers of myocardial diastolic native T1 maps with artifacts. Vertical axis stands for the numbers of diastolic native T1 maps with artifacts, horizontal axis stands for the presented highest heart rate during scan, below are the patient numbers at different heart rate levels

For the 30 patients with “persistent” AF, there were 378 native T1 maps and 378 post-contrast T1 maps (27 patients × 3 slices × 4 (diastole/systole + fixed/HRD sampling scheme combination) + 3 patients × 3 slices × 6 (1^st^ CMR: diastole/systole + fixed/HRD sampling scheme combination = 4; 2^nd^ CMR: diastole/systole + HRD sampling scheme combination = 2)). 599 segments in 113 native T1 maps and 592 segments in 111 post-contrast T1 maps were excluded due to the presence of artifacts. The artifacts we encountered mainly were off resonance related dark banding artifact and mistrigger related motion artifact (Fig. 2). The dark banding artifact were presented evenly in diastolic and systolic images, although the local shimming were applied and different center frequencies were tried, 35 segments (0.99 %) were with identified banding artifact ultimately. The mistrigger related motion artifacts were more commonly found in diastolic images than systolic images. In the native T1 maps with motion artifacts, 542 segments (15.36 %) of 100 T1 maps (13.23 %) were diastolic images; in the post-contrast T1 maps with artifacts, 531 segments (15.05 %) of 97 T1 maps (12.83 %) were diastolic images. Overall, there were more assessable T1 maps in systole than diastole with significant differences (*χ*^2^ = 201.003, *p* < 0.01). Most motion artifacts were presented in patients with moderately rapid heart rate and/or with severe heart rate fluctuation (Fig. 3). In very rapid heart rate (e.g. 130 bpm), the system default diastolic TD was close to the systolic TD of 0 ms, therefore we obtained two series of similar “systolic” T1 maps. Consequently, very fast heart rate was observed with relatively less number of inferior quality images compared to moderate rapid heart rate in diastolic T1 maps (Fig. 4).

The myocardial native T1 times and ECV generated from HRD sampling scheme T1 maps were greater than fixed sampling scheme T1 maps either in diastolic or systolic phase with statistical significance (all *p* < 0.05). The myocardial native T1 times generated from diastolic T1 maps were longer than systolic T1 maps either in fixed or in HRD scheme T1 maps with statistical significance (all *p* < 0.05), the ECV generated from diastole was larger than in systole, either in fixed or HRD sampling scheme without statistical significance (all *p* > 0.05). In the pre-contrast LV blood T1* maps, blood T1 times generated from diastolic phase were longer than systolic phase, while in the post-contrast LV blood T1* maps, the blood T1 times from diastolic phase were shorter than systolic phase. Regarding the comparison between fixed and HRD scheme, blood T1 times generated from fixed scheme were longer than HRD scheme in pre-contrast; In post-contrast CMR, the blood T1 from fixed scheme were shorter than HRD scheme (nearly half of these differences were statistical significant). The LV overall native T1 times, post-contrast T1 times and ECV of myocardium, pre-/post-contrast blood T1 times generated from different diastolic/systolic and fixed/HRD scheme combination were compared and are shown in Table 3.

**Table 3:** Comparison of pre-/post-contrast myocardium and blood T1 times and ECV between different diastolic/systolic and fixed/HRD scheme T1 maps in patients with persistent AF

	1	2	3	4	*P* value
	Diastole with fixed scheme	Systole with fixed scheme	Diastole with HRD scheme	Systole with HRD scheme	
Overall LV myocardium native T1 times (ms)	1272.76 ± 39.30	1263.29 ± 40.71	1311.00 ± 44.51	1304.83 ± 52.01	1 vs. 2: <0.01
					3 vs. 4: <0.05
					1 vs. 3: <0.01
					2 vs. 4: <0.01
Overall LV myocardium post-contrast T1 times (ms)	543.42 ± 57.04	551.07 ± 57.49	571.37 ± 66.16	588.94 ± 64.31	1 vs. 2: <0.05
					3 vs. 4: >0.05
					1 vs. 3: >0.05
					2 vs. 4: <0.05
Overall LV pre-contrast blood T1 times (ms)	1819.40 ± 136.03	1797.49 ± 132.23	1796.32 ± 132.02	1766.30 ± 133.51	1 vs. 2: >0.05
					3 vs. 4: >0.05
					1 vs. 3: >0.05
					2 vs. 4: >0.05
Overall LV post-contrast blood T1 times (ms)	359.61 ± 53.34	366.06 ± 52.73	393.03 ± 61.73	400.18 ± 64.13	1 vs. 2: <0.01
					3 vs. 4: >0.05
					1 vs. 3: <0.05
					2 vs. 4: <0.05
Overall LV ECV (%)	26.70 ± 2.45	26.41 ± 2.73	28.02 ± 3.48	27.18 ± 3.36	1 vs. 2: >0.05
					3 vs. 4: >0.05
					1 vs. 3: <0.05
					2 vs. 4: <0.05

Data are presented in mean ± standard deviation. *ECV* extracellular volume, *HRD* heart-rate-dependent, *AF* atrial fibrillation, *LV* left ventricle

Three patients underwent CMR 2 times, before and after electric cardioversion. Patient 1’s heart rate during 1^st^ CMR ranged 69–116 bpm, HRD scheme was 5(11)3/4(6)3(6)2; after electric cardioversion, the patient’s heart rate during 2^nd^ CMR ranged 77–80 bpm, scheme was 5(5)3/4(2)3(2)2. Patient 2’s heart rate during 1^st^ CMR ranged 64–87 bpm, HRD scheme was 5(7)3/4(3)3(3)2; the patient’s heart rate during 2^nd^ CMR ranged 62–65 bpm, scheme was 5(4)3/4(1)3(1)2. Patient 3’s heart rate during 1^st^ CMR ranged 80–101 bpm, HRD was 5(9)3/4(5)3(5)2; the patient’s heart rate during 2^nd^ CMR ranged 75–77 bpm, scheme was 5(5)3/4(2)3(2)2. For all the 3 patients, either in the diastolic or systolic T1 maps, there were significant differences of per-segment native T1 times and ECV between 1^st^ CMR fixed scheme and HRD scheme, and between 1^st^ CMR fixed scheme and 2^nd^ CMR HRD scheme (all *p* <0.01), but no significant differences of per-segment native T1 times and ECV between 1^st^ CMR HRD scheme and 2^nd^ CMR HRD scheme (*p* >0.05, Table 4).

**Table 4:** Comparison of per-segment native T1 times and ECV in patients before/after electric cardioversion

			Patient 1		Patient 2		Patient 3	
				*P* value		*P* value		*P* value
Diastole	A: fixed	Native T1 (ms)	1294.67 ± 16.50	A vs. B: <0.01	1233.83 ± 25.09	A vs. B: <0.05	1268.80 ± 34.85	A vs. B: <0.01
	B: 1^st^ HRD	Native T1 (ms)	1353.30 ± 36.55	B vs. C: >0.05	1241.17 ± 16.08	B vs. C: >0.05	1289.57 ± 13.29	B vs. C: >0.05
	C: 2^nd^ HRD	Native T1 (ms)	1342.60 ± 18.12	A vs. C: <0.01	1245.07 ± 11.98	A vs. C: <0.05	1289.07 ± 16.05	A vs. C: <0.01
	A: fixed	ECV (%)	23.12 ± 0.22	A vs. B: <0.01	22.28 ± 1.00	A vs. B: <0.01	27.25 ± 0.34	A vs. B: <0.01
	B: 1^st^ HRD	ECV (%)	25.51 ± 0.88	B vs. C: >0.05	24.96 ± 0.24	B vs. C: >0.05	28.49 ± 0.37	B vs. C: >0.05
	C: 2^nd^ HRD	ECV (%)	25.47 ± 1.14	A vs. C: <0.01	25.60 ± 0.97	A vs. C: <0.01	28.50 ± 0.21	A vs. C: <0.01
Systole	A: fixed	Native T1 (ms)	1283.30 ± 34.76	A vs. B: <0.01	1209.90 ± 33.24	A vs. B: <0.01	1249.73 ± 39.49	A vs. B: <0.01
	B: 1^st^ HRD	Native T1 (ms)	1345.1 ± 23.40	B vs. C: >0.05	1246.37 ± 33.92	B vs. C: >0.05	1274.70 ± 17.68	B vs. C: >0.05
	C: 2^nd^ HRD	Native T1 (ms)	1339.57 ± 25.75	A vs. C: <0.01	1250.80 ± 36.09	A vs. C: <0.01	1256.10 ± 17.91	A vs. C: <0.05
	A: fixed	ECV (%)	23.57 ± 0.62	A vs. B: <0.05	22.86 ± 2.58	A vs. B: <0.05	27.01 ± 1.50	A vs. B: <0.05
	B: 1^st^ HRD	ECV (%)	24.41 ± 1.74	B vs. C: >0.05	23.74 ± 0.56	B vs. C: >0.05	27.13 ± 0.65	B vs. C: >0.05
	C: 2^nd^ HRD	ECV (%)	24.76 ± 0.52	A vs. C: <0.05	23.56 ± 1.21	A vs. C: <0.05	27.14 ± 0.90	A vs. C: <0.05

Data are presented in mean ± standard deviation. *ECV* extracellular volume, *HRD* heart-rate-dependent

## Discussion

The main findings of this study include: the HRD sampling scheme can avoid the underestimation of T1 times in the setting of rapid ventricle rate either in sinus rhythm or arrhythmia; although the native T1 times/ECV of myocardium in diastole are greater than in systole, to obtain data at systole can improve the image quality by decreasing the chance of mistriggering in patients with AF. Finally, a subset of patients with repeated T1 mapping demonstrated the effectiveness of systolic HRD sampling scheme in patients with AF. In addition, to the best of our knowledge, this is the first study using native T1 and ECV assessing LV myocardium in patients with AF before catheter ablation. Compared to healthy volunteers, the patients with AF showed greater native T1 times and ECV, which may be an indicator for myocardial fibrosis. Our findings are in line with other studies using other methods or animal study [16, 17].

### Validation of T1 mapping in HRD sampling scheme

Incomplete inversion recovery may lead to an underestimation of T1 times. In patients with higher heart rate, the R-R interval shortened, and the fixed sampling scheme of 5(3)3/4(1)3(1)2 does not provide enough time for the magnetization to get back to the equilibrium. Increasing the number of heartbeats after T1 inversion recovery can avoid underestimation of T1 times. In our study, we tested the feasibility and accuracy of HRD sampling scheme in healthy volunteers and patients with AF with repeated CMRs. In volunteers and patients with AF, the T1 times and ECV generated from fixed sampling scheme were smaller than HRD sampling scheme, and repeated T1 mapping demonstrated that the HRD sampling scheme can more accurately estimate the T1 times than the fixed sampling scheme. In volunteer 1, the heartbeats number of T1 inversion recovery increased in a small range (pre-contrast: 3 to 4, post-contrast: 1 to 2), although the myocardial native T1 times and ECV generated from 1^st^ fixed sampling scheme were smaller than 1^st^ HRD and 2^nd^ fixed sampling scheme, the differences did not reach statistical significance. Volunteer 2’s heartbeats number of T1 inversion recovery increased in a larger range (3 to 7 and 1 to 3), the differences of myocardial native T1 times and ECV between 1^st^ fixed sampling scheme and 1^st^ HRD/2^nd^ CMR fixed sampling scheme reached statistical significance. In the 3 patients with AF, all the heartbeats number of T1 inversion recovery increased with a large range (3 to 11, 7, 9 in the pre-contrast, and 1 to 6, 3, 5 in the post-contrast respectively). The myocardial native T1 times and ECV of 1^st^ fixed sampling scheme were smaller than 1^st^ and 2^nd^ HRD sampling scheme with statistical significance. The impact of the HRD sampling scheme was more prominent for higher heart rates. Thereby, HRD sampling scheme is practical for rapid ventricle rate either in sinus rhythm or arrhythmia.

### Impact of cardiac cycle on T1 mapping

In healthy volunteers and patients with AF, both for fixed and HRD sampling scheme, the myocardial native T1 times and ECV in diastole were greater than in systole. Our findings concur in general with some previous studies [13, 18, 19]. The differences of myocardial T1 times between diastole and systole can be explained by partial-volume effect on diastole [13] and/or reduced intra-myocardial blood volume contamination in systole [18]. Nevertheless, the mean differences of native T1 times (healthy volunteer: 16.67 ms; patients with AF: 9.47 ms with fixed scheme and 6.17 ms with HRD scheme) and ECV (healthy volunteer: 0.51 %; patients with AF: 0.29 % with fixed scheme and 0.84 % with HRD scheme) were small and might not be clinically relevant on an individual basis. Even so, it is recommended to obtain and compare T1 maps in the same cardiac phase to avoid potential bias [5].

Although apparent myocardial native T1 times and ECV in systole were smaller than in diastole, more images in systole were evaluable than in diastole. In line with other studies [13, 18, 20], our results revealed that T1 mapping acquisition in systole increases the number of evaluable images and segments both in healthy volunteers and patients with AF. At higher heart rates, the motion-free time in diastole shortens more than in systole. In patients with AF, although the R-R interval varies in each cardiac cycle, the variation in systole is smaller than in diastole [11, 13] (Fig. 2). Therefore, the acquisition in systole resulted in more evaluable images. But in case of lower ventricular rate, at mid to end diastole, cardiac motion is relatively minor, and even in patients with AF and low heart rates, diastolic images were presented with less artifacts [13] (Fig. 3).

### Diffuse ventricular fibrosis in patients with AF

Except the main finding of our study, our results also revealed that the myocardial native T1 times and ECV of patients with AF were greater than in healthy volunteers. The cardiac profibrotic microenvironment in AF is unlikely to be strictly limited to the atria, and the ventricular myocardium is also likely to be affected, ventricular fibrotic changes are more pronounced in AF patients than in subjects with sinus rhythm [1]. Although the effect of ventricular fibrosis in the pathogenesis of AF is controversial, recent study found that diffuse ventricular fibrosis is an independently predictor of AF recurrence after ablation therapy [3]. T1 mapping and ECV allow quantitative assessment of diffuse myocardial characteristics fibrosis, which was only possible with biopsy in the past. Ling et al. demonstrated diffuse ventricular fibrosis in AF by employing post-contrast T1 mapping [2]. Furthermore, our study demonstrated diffuse ventricular fibrosis in AF using the reliable parameter of ECV. This technique provides a new method for investigating the implication of ventricular fibrosis in AF.

### Limitations

The number of participants included in this study is relatively small, especially the number of participants who underwent the CMR twice. Since the repeated CMRs can test the efficiency of the HRD sampling scheme, more participants need to be enrolled in a future study. The HRD sequence used in this study is relatively old version, the inversion time is expressed as heartbeat, now a more advanced sequence with seconds as unit is widely accepted by T1 mapping research community. Due to the sequence version’s limitations, we are not able to test this sequence in our study. However, it’s suggested that we utilize this advanced version of sequence in our future study. The operation of the HRD sampling scheme is relatively complicated, and the impact of HRD sampling scheme is more prominent in higher heart rate, therefore further investigation is needed to simplify the operation under the condition of keeping the feature of HRD sampling scheme. Finally, we did not perform the intra- and inter-observer variability, although a good agreement of intra- and inter-observer variability has been demonstrated by other studies [13, 20].

## Conclusions

The heart-rate-dependent sampling scheme is helpful for avoiding underestimation of T1 times, especially in subjects with higher heart rates. Systolic T1 mapping yields shorter myocardial native T1 times and ECV, but the mean differences were small and, importantly, with the benefit of improvement in data quality in subjects with irregular and rapid heart rate. Together with these findings, systolic MOLLI T1 mapping with a heart-rate-dependent sampling scheme is feasible and its clinical application can be extended to patients with atrial fibrillation.

## Abbreviations

AF: atrial fibrillation
ECV: extracellular volume fraction
MOLLI: Modified Look-Locker Inversion Recovery
HRD: heart-rate-dependent
CMR: cardiovascular magnetic resonance
TD: trigger delay
LGE: late gadolinium enhancement
LV: left-ventricular
